# Oestradiol and sex hormone-binding globulin in premenopausal and post-menopausal meat-eaters, vegetarians and vegans

**DOI:** 10.1038/sj.bjc.6690546

**Published:** 1999-07

**Authors:** H V Thomas, G K Davey, T J Key

**Affiliations:** Imperial Cancer Research Fund Cancer Epidemiology Unit, Gibson Building, Radcliffe Infirmary, Oxford, OX2 6HE, UK; Department of Psychological Medicine, University of Wales College of Medicine, Heath Park, Cardiff, CF4 4XN, UK

**Keywords:** vegetarian, vegan, oestradiol, sex hormone-binding globulin, body mass index, breast cancer

## Abstract

Endogenous oestradiol is strongly associated with breast cancer risk but its determinants are poorly understood. To test the hypothesis that vegetarians have lower plasma oestradiol and higher sex hormone-binding globulin (SHBG) than meat-eaters we assayed samples from 640 premenopausal women (153 meat-eaters, 382 vegetarians, 105 vegans) and 457 post-menopausal women (223 meat-eaters, 196 vegetarians, 38 vegans). Vegetarians and vegans had lower mean body mass indices (BMI) and lower plasma cholesterol concentrations than meat-eaters, but there were no statistically significant differences between meat-eaters, vegetarians and vegans in pre- or post-menopausal plasma concentrations of oestradiol or SHBG. Before adjusting for BMI there were small differences in the direction expected, with the vegetarians and vegans having higher SHBG and lower oestradiol (more noticeable amongst post-menopausal women) than the meat-eaters. These small differences were essentially eliminated by adjusting for BMI. Thus this study implies that the relatively low BMI of vegetarians and vegans does cause small changes in SHBG and in post-menopausal oestradiol, but that the composition of vegetarian diets may not have any additional effects on these hormones. © 1999 Cancer Research Campaign


British Journal of Cancer (1999) 80(9), 1470–1475
© 1999 Cancer Research Campaign
Article no. bjoc.1998.0546

### Oestradiol and sex hormone-binding globulin in premenopausal and post-menopausal meat-eaters, vegetarians and vegans

**HV Thomas[1,2], GK Davey[1] and TJ Key[1]**

[1]Imperial Cancer Research Fund Cancer Epidemiology Unit, Gibson Building, Radcliffe Infirmary, Oxford OX2 6HE, UK; [2]Department of Psychological Medicine, University of Wales College of Medicine, Heath Park, Cardiff CF4 4XN, UK

**Summary** Endogenous oestradiol is strongly associated with breast cancer risk but its determinants are poorly understood. To test the hypothesis that vegetarians have lower plasma oestradiol and higher sex hormone-binding globulin (SHBG) than meat-eaters we assayed samples from 640 premenopausal women (153 meat-eaters, 382 vegetarians, 105 vegans) and 457 post-menopausal women (223 meat-eaters, 196 vegetarians, 38 vegans). Vegetarians and vegans had lower mean body mass indices (BMI) and lower plasma cholesterol concentrations than meat-eaters, but there were no statistically significant differences between meat-eaters, vegetarians and vegans in pre- or post-menopausal plasma concentrations of oestradiol or SHBG. Before adjusting for BMI there were small differences in the direction expected, with the vegetarians and vegans having higher SHBG and lower oestradiol (more noticeable amongst post-menopausal women) than the meat-eaters. These small differences were essentially eliminated by adjusting for BMI. Thus this study implies that the relatively low BMI of vegetarians and vegans does cause small changes in SHBG and in post-menopausal oestradiol, but that the composition of vegetarian diets may not have any additional effects on these hormones.

**Keywords:** vegetarian; vegan; oestradiol; sex hormone-binding globulin; body mass index; breast cancer

Epidemiological and biological data suggest that oestradiol may be an important determinant of breast cancer risk (Pike et al, 1993), and there is now substantial evidence from several prospective studies of a strong association between plasma oestradiol and breast cancer risk in post-menopausal women (Thomas et al, 1997*a*; Hankinson et al, 1998). It is the oestradiol that is not bound to sex hormone-binding globulin (SHBG) that is thought to be bioavailable (Siiteri et al, 1981), so that relatively high concentrations of SHBG may reduce breast cancer risk. Obesity causes an increase in oestradiol concentration in post-menopausal women and a decrease in SHBG concentration in both pre- and post-menopausal women (Potischman et al, 1996; Thomas et al, 1997*b*), but the possible effects of dietary composition on oestradiol and SHBG are not well understood. Women in countries with low breast cancer rates and low serum oestradiol concentrations typically have a plant-based diet, low in animal products (e.g. Key et al, 1990), and it has been suggested that a vegetarian diet may decrease endogenous concentrations of oestradiol and increase concentrations of SHBG (Armstrong et al, 1981; Goldin et al, 1982; Gray et al, 1982; Shultz and Leklem, 1983; Shultz et al, 1987; Fentiman et al, 1988; Adlercreutz et al, 1989; Barbosa et al, 1990; Persky et al, 1992). However, the results of these studies were not consistent and none of them included more than 50 vegetarian women.

We report a study to test the hypotheses that a vegetarian or vegan diet is associated with a relatively low plasma concentration of oestradiol and a relatively high concentration of SHBG in both pre- and post-menopausal women. The study involved 640 premenopausal and 457 post-menopausal British women and is by far the largest to date to investigate differences in endogenous concentrations of oestradiol and SHBG between meat-eaters and vegetarians. It is also the first study to report on oestradiol and SHBG concentrations in a large sample of vegan women.

*Received 20 October 1998*
*Revised 23 November 1998*
*Accepted 23 November 1998*

*Correspondence to:* TJ Key

### SUBJECTS AND METHODS

### Study subjects and data collection

During 1994 and 1995, 16 790 women aged 20 years and above and living in the UK were recruited into a prospective study, part of the European Prospective Investigation into Nutrition and Cancer (Riboli and Kaaks, 1997). Participants were recruited through vegetarian and health food magazines, the Vegetarian Society and the Vegan Society, and from the friends and relatives of participants; the subjects recruited by this last method included many meat-eaters as well as vegetarians and vegans.

All participants completed a questionnaire which included details of age, height, weight, reproductive history, menopausal status, use of oral contraceptives and other hormonal therapy, and consumption of meat, fish, dairy products and eggs. Participants were classified as meat-eaters if they recorded that they ate any meat (including poultry), as vegetarians if they recorded that they did not eat meat or fish but did eat dairy products and/or eggs, and as vegans if they recorded that they did not eat meat, fish, dairy products or eggs. A 30 ml blood sample was obtained from each volunteer and sent through the post to the laboratory. Plasma was prepared, divided into 2-ml aliquots and stored at − 50°C.

The present analyses include 640 premenopausal women who were aged 20–44, reported having ten or more menstrual periods in the last 12 months and had a most recent menstrual cycle less than 40 days long. Of these 640 women, 153 were meat-eaters, 382 were vegetarians and 105 were vegans. The analyses also include 457 post-menopausal women who were aged 55–85 and reported having no menstrual periods in the last 12 months. Of these 457 women, 223 were meat-eaters, 196 were vegetarians and 38 were vegans.

Women were excluded if they could not be classified into one of the three dietary groups, if they were using any exogenous sex hormones or were pregnant at the time of blood collection, if their number of menstrual periods in the last 12 months was unknown, if the day of their menstrual cycle on which their blood sample was collected was unknown, if they had undergone a hysterectomy and were aged less than 60 or had undergone an oophorectomy, or if they reported ever being diagnosed with cancer of any type.

### Measurement of plasma concentrations of oestradiol, SHBG and lipids

Plasma samples were randomly assorted into assay batches ensuring that the proportions of meat-eaters, vegetarians and vegans in each batch were identical to the proportions of each diet group in the total number of women available for analysis. The samples were then randomly ordered within each batch. Assays were performed without knowledge of the dietary classification of the subjects involved. All measurements of lipids and of premenopausal concentrations of oestradiol and SHBG were carried out using singleton samples; post-menopausal concentrations of oestradiol and SHBG were measured in duplicate. The plasma samples were thawed and refrozen on three occasions: for the oestradiol assay, the SHBG assay and for the lipid measurements in that order.

Premenopausal plasma oestradiol concentrations were measured by a heterogeneous competitive magnetic separation assay (Bayer plc, Berkshire, UK) at the Biochemical Endocrinology laboratory at the Radcliffe Infirmary, Oxford. The percentage coefficent of variation (% CV), which incorporates both intra-assay and inter-assay variation, was 4.8% at an expected concentration range of 325–444 pmol $l^{-1}$. The lowest detectable concentration was 37 pmol $l^{-1}$.

Post-menopausal oestradiol concentrations were measured at the Biochemical Endocrinology laboratory at the Royal Marsden Hospital, London by radioimmunoassay after ether extraction (Dowsett et al, 1987). Intra- and inter-assay % CVs were 16.3% and 21.8% at an expected concentration range of 15–30 pmol $l^{-1}$. The lowest detectable concentration was 3.0 pmol $l^{-1}$.

Pre- and post-menopausal plasma concentrations of SHBG were measured using a non-competitive liquid-phase IRMA kit (Farmos Diagnostica, Oulansalo, Finland) at the Biochemical Endocrinology laboratories at the Radcliffe Infirmary and at the Royal Marsden Hospital respectively. The % CVs were 6.2% and 4.1%, respectively, at an expected concentration range of 74–112 nmol $l^{-1}$. The lowest detectable concentration was 6.25 nmol $l^{-1}$.

Plasma lipid concentrations were measured at the Clinical Biochemistry laboratory, Addenbrooke's Hospital, Cambridge. Concentrations of total cholesterol and triglycerides were measured by automated enzymatic procedures using reagents supplied by Bayer, concentrations of high-density lipoprotein (HDL) cholesterol were measured by a similar method after pretreatment with a precipitant supplied by Boehringer Mannheim. Low-density lipoprotein (LDL) cholesterol was calculated according to the Friedewald formula as total cholesterol

**Table 1** Characteristics of the three dietary groups

| Variable | Meat-eaters Mean (95% CI) | Vegetarians Mean (95% CI) | Vegans Mean (95% CI) |
|---|---|---|---|
| *Premenopausal* | | | |
| *n* | 153 | 382 | 105 |
| Age at recruitment (years) | 37.6 (36.7–38.4) | 35.5 *(34.9–36.2) | 32.5* (31.3–33.8) |
| Age at menarche (years)[a] | 12.8 (12.6–13.0) | 12.8 (12.6–12.9) | 12.7 (12.5–13.0) |
| Length of menstrual cycle (days) | 28.1 (27.4–28.7) | 27.9 (27.6–28.3) | 27.9 (27.2–28.6) |
| Weight (kg)[b] | 64.4 (62.6–66.2) | 60.2* (59.3–61.2) | 59.7* (58.0–61.4) |
| BMI (kg $m^{-2}$)[b] | 23.5 (22.9–24.2) | 22.3* (22.0–22.6) | 22.0* (21.4–22.6) |
| Plasma cholesterol (mmol $l^{-1}$) | 4.18 (4.06–4.29) | 3.85* (3.78–3.92) | 3.55* (3.43–3.67) |
| LDL cholesterol (mmol $l^{-1}$)[c] | 2.62 (2.52–2.73) | 2.29* (2.23–2.35) | 2.03* (1.94–2.13) |
| HDL cholesterol (mmol $l^{-1}$)[d] | 1.30 (1.26–1.35) | 1.31 (1.29–1.34) | 1.28 (1.23–1.34) |
| *Postmenopausal* | | | |
| *n* | 223 | 196 | 38 |
| Age at recruitment (years) | 64.0 (63.3–64.7) | 63.8 (63.0–64.5) | 63.1 (61.6–64.6) |
| Age at menarche (years)[e] | 12.9 (12.7–13.1) | 13.1 (12.9–13.4) | 13.2 (12.6–13.8) |
| Age at menopause (years)[f] | 49.9 (49.3–50.6) | 49.9 (49.2–50.5) | 48.8 (47.2–50.5) |
| Years past menopause[f] | 13.7 (12.7–14.7) | 13.4 (12.2–14.5) | 14.3 (11.9–16.6) |
| Weight (kg)[g] | 65.4 (63.7–67.1) | 60.7* (59.3–62.1) | 60.9 (57.3–64.5) |
| Body mass index (kg $m^{-2}$)[h] | 24.5 (23.9–25.1) | 22.7* (22.2–23.2) | 23.0 (21.7–24.3) |
| Plasma cholesterol (mmol $l^{-1}$)[i] | 5.36 (5.25–5.48) | 5.01* (4.90–5.13) | 4.55* (4.28–4.81) |
| LDL cholesterol (mmol $l^{-1}$)[i] | 3.58 (3.48–3.69) | 3.21* (3.11–3.32) | 2.79* (2.57–3.02) |
| HDL cholesterol (mmol $l^{-1}$)[i] | 1.27 (1.22–1.31) | 1.30 (1.26–1.34) | 1.32 (1.22–1.42) |

[a]Data for 381 vegetarians, 104 vegans. [b]Data for 149 meat-eaters, 374 vegetarians. [c]Low-density lipoprotein cholesterol. [d]High-density lipoprotein cholesterol. [e]Data for 219 meat-eaters, 193 vegetarians, 37 vegans. [f]Data for 174 meat-eaters, 142 vegetarians, 32 vegans. [g]Data for 213 meat-eaters, 190 vegetarians, 37 vegans. [h]Data for 211 meat-eaters, 190 vegetarians, 37 vegans. [i]Data for 221 meat-eaters, 195 vegetarians. *Difference between mean with meat-eaters as comparison group, $P < 0.05$.

© 1999 Cancer Research Campaign 

**Table 2** Plasma concentrations of oestradiol and SHBG in the three dietary groups

| Hormone (units) | Meat-eaters<br>*n* Mean (95%CI) | Vegetarians<br>*n* Mean (95%CI) | Vegans<br>*n* Mean (95%CI) | *P*- value |
|---|---|---|---|---|
| *Premenopausal* | | | | |
| Oestradiol (pmol l$^{-1}$) | | | | |
| Whole cycle | 153 357 (330–386) | 382 351 (334–369) | 105 351 (320–386) | 0.95 |
| **Adjusted for BMI** | **360 (332–389)** | **350 (333–367)** | **349 (318–384)** | **0.83** |
| Follicular phase[a] | 59 239 (210–272) | 139 241 (222–263) | 35 228 (193–270) | 0.85 |
| Midcycle[b] | 22 592 (467–749) | 59 639 (553–738) | 19 731 (566–942) | 0.51 |
| Luteal phase[c] | 72 412 (371–457) | 184 384 (359–410) | 51 382 (337–432) | 0.52 |
| SHBG (nmol l$^{-1}$) | 153 45.0 (42.0–48.1) | 382 46.2 (44.3–48.2) | 105 48.3 (44.5–52.3) | 0.45 |
| **Adjusted for BMI** | **46.8 (43.9–49.9)** | **45.5 (43.7–47.4)** | **47.3 (43.8–51.0)** | **0.61** |
| *Postmenopausal* | | | | |
| Oestradiol (pmol l$^{-1}$) | 223 18.7 (17.3–20.2) | 196 18.0 (16.8–19.3) | 38 17.5 (14.9–20.4) | 0.69 |
| **Adjusted for BMI** | **17.7 (17.1–18.3)** | **19.1 (17.8–20.4)** | **18.3 (16.9–19.9)** | **0.32** |
| SHBG (nmol l$^{-1}$) | 223 43.3 (40.7–46.0) | 196 46.0 (42.9–49.4) | 38 48.4 (42.7–54.8) | 0.25 |
| **Adjusted for BMI** | **45.4 (44.1–46.8)** | **43.7 (41.2–46.4)** | **47.4 (44.3–50.8)** | **0.46** |

Values are geometric means. All premenopausal concentrations are adjusted for age in categories of 20–29, 30–34, 35–39 and 40–44 years old. Premenopausal concentrations of oestradiol are adjusted for day of menstrual cycle in categories of 0–2, 3–5, 6–7, 8–10, 11–13, 14–17, 18–21, 22–24 and 25+ days before next menstrual period; premenopausal concentrations of SHBG are adjusted in categories of 0–2, 3–13, 14–17, 18–21 and 22+ days before next menstrual period. All *P*-values are for differences between means. [a]18+ days before next menstrual period. [b]14–17 days before next menstrual period. [c]0–13 days before next menstrual period.

minus (triglycerides ÷ 2.19) minus HDL cholesterol (Friedewald et al, 1972). The inter-assay % CVs were calculated from several months of inter-batch analysis. The % CV at a cholesterol concentration of 2.6 mmol l$^{-1}$ was 1.4%, at a triglyceride concentration of 2.7 mmol l$^{-1}$ was 1.1%, and at an HDL cholesterol concentration of 1.3 mmol l$^{-1}$ was 1.3%. Lipid measurements were not made for three women (all post-menopausal) due to insufficient plasma.

### Statistical analyses

Plasma concentrations of oestradiol and SHBG were logarithmically transformed to produce approximately normal distributions and the mean hormone concentrations presented are geometric means. Associations with hormone concentrations and with diet were investigated for the following variables: age at recruitment, age at menarche, length of menstrual cycle during which the blood sample was donated, age at menopause, number of years past menopause, previous hysterectomy (yes/no), parity (parous/nulliparous), weight, body mass index (BMI), total plasma cholesterol concentration, LDL cholesterol concentration, HDL cholesterol concentration and the hour of the day of blood sample collection. The associations between continuous variables and the three dietary groups were examined using one-way analysis of variance; the significance of the difference between means of any two of the dietary groups was assessed by the Bonferroni test. The frequency distributions of categorical variables among the three dietary groups were investigated using the $\chi^2$ test; statistically significant differences in these frequency distributions were detected using either the likelihood ratio test or the Mantel–Haenszel test for linear association. The associations between the plasma hormone concentrations and other variables were explored using Pearson and partial correlation coefficients and analysis of variance and covariance.

For all analyses involving the premenopausal subjects, oestradiol concentration was adjusted for day of menstrual cycle split into nine categories (0–2, 3–5, 6–7, 8–10, 11–13, 14–17, 18–21, 22–24 and 25+ days before next menstrual period) and SHBG was adjusted for day of menstrual cycle split into five categories (0–2, 3–13, 14–17, 18–21 and 22+ days before next menstrual period). Further adjustments were made separately for each of the following: age, BMI, parity and hour of day of blood sample collection. For analyses involving the post-menopausal subjects, adjustments were made separately for each of the following: age, number of years past menopause, BMI, parity and hour of day of blood sample collection. Instead of adjusting for previous hysterectomy, the comparison of mean hormone concentrations between the three dietary groups was repeated excluding women who had either previously undergone a hysterectomy ($n = 34$) or for whom this information was missing ($n = 2$).

All statistical tests were considered significant at $P < 0.05$. Two-sided $P$-values are quoted. All statistical analyses were performed using SPSS (SPSS Inc., Chicago, USA).

### RESULTS

### Characteristics of the three dietary groups

Table 1 displays the descriptive characteristics of the three dietary groups among the 640 premenopausal women and the 457 postmenopausal women in this study. Amongst the premenopausal women, the meat-eaters were, on average, approximately 2 years older than the vegetarians, who in turn were 3 years older than the vegans. The meat-eaters were significantly heavier than the vegetarians and vegans and had a 5% higher BMI than the vegetarians and a 7% higher BMI than the vegans. Total cholesterol concentration was 8% higher in the meat-eaters than in the vegetarians and 17% higher than in the vegans, LDL cholesterol concentration was 13% higher in the meat-eaters than in the vegetarians and 30% higher than in the vegans, whilst the mean HDL cholesterol concentration was almost identical in the three dietary groups. The three dietary groups were almost identical in age at menarche and length of menstrual cycle.

 © 1999 Cancer Research Campaign

**Table 3** Studies of oestradiol and SHBG in vegetarians and non-vegetarians

| Reference | Diet | *n* | Cycle phase | Oestradiol (pmol $l^{-1}$) | SHBG (nmol $l^{-1}$) | BMI (kg $m^{-2}$) |
|---|---|---|---|---|---|---|
| *Premenopausal* | | | | | | |
| Goldin et al 1982 | Vegetarian | 10 | Follicular | 260 | | 22.6 |
| | Non-vegetarian | 10 | | 320 | | 22.7 |
| Gray et al 1982 | Vegetarian | 23 | Follicular | 220 | | 20.9 |
| | Non-vegetarian | 26 | | 231 | | 21.6 |
| | Vegetarian | 23 | Luteal | 485 | | |
| | Non-vegetarian | 26 | | 430 | | |
| Shultz and Leklem 1983 | Vegetarian | 14 | Luteal | 477 | | 22.0 |
| | Non-vegetarian | 9 | | 684 | | 23.3 |
| Shultz et al 1987 | Vegetarian | 10 | Luteal | 559 | | 21.1 |
| | Non-vegetarian | 10 | | 622 | | 22.3 |
| Fentiman et al 1988[a] | Vegetarian | 25 | Whole | | 59.9 | 21.6 |
| | Non-vegetarian | 21 | cycle | | 62.0 | 23.1 |
| Persky et al 1992 | Vegetarian | 34 | Follicular | 367 | | 22.8 |
| | Non-vegetarian | 39 | | 260 | | 22.2 |
| | Vegetarian | 33 | Luteal | 412 | | |
| | Non-vegetarian | 31 | | 519 | | |
| Present study[b] | Vegan | 105 | Whole | 351 | 48.3 | 22.0 |
| | Vegetarian | 382 | cycle | 351 | 46.2 | 22.3 |
| | Non-vegetarian | 153 | | 357 | 45.0 | 23.5 |
| *Postmenopausal* | | | | | | |
| Armstrong et al 1981 | Vegetarian | 43 | | 66.4 | 23.1 | |
| | Non-vegetarian | 44 | | 57.4 | 24.0 | |
| Adlercreutz et al 1989 | Vegetarian | 9 | | 37.7 | 27.2 | |
| | Non-vegetarian | 10 | | 45.6 | 22.8 | |
| Barbosa et al 1990 | Vegetarian | 12 | | 13.1 | 51.3 | 22.5 |
| | Non-vegetarian | 12 | | 23.8 | 58.1 | 24.7 |
| Present study[b] | Vegan | 38 | | 17.5 | 48.4 | 23.0 |
| | Vegetarian | 196 | | 18.0 | 46.0 | 22.7 |
| | Non-vegetarian | 223 | | 18.7 | 43.3 | 24.5 |

Values of oestradiol, SHBG and BMI are mean values. [a]Approximation of mean BMI derived from the reported mean weight and mean height for the two dietary groups. [b]Mean values unadjusted for BMI

Amongst the post-menopausal women, the three dietary groups were similar in age at recruitment and age at menarche. The vegans were, on average, approximately 1 year younger at menopause than the vegetarians and meat-eaters and, accordingly, were approximately 1 year further past the menopause. The meat-eaters were approximately 8% heavier than the vegetarians and vegans, and had a statistically significant higher BMI than the vegetarians. Total cholesterol concentration was 8% higher in the meat-eaters than in the vegetarians and 20% higher than in the vegans, LDL cholesterol concentration was 13% higher in the meat-eaters than in the vegetarians and 29% higher than in the vegans, whilst the mean HDL cholesterol concentration was almost identical in the three dietary groups.

There was a highly significant trend in parity across the three dietary groups in both the premenopausal and post-menopausal women. Amongst the premenopausal women, 58.8% of meat-eaters, 45.8% of vegetarians and 21.0% of vegans were parous ($P < 0.001$), whilst among the post-menopausal women these figures were 83.0%, 75.0% and 65.8% respectively ($P = 0.006$). Amongst the post-menopausal women, the proportion of meat-eaters who had previously undergone a hysterectomy was approximately double the proportion of vegetarians who had had a hysterectomy (10.4% vs 5.1%, $P = 0.05$). When stratified by parity, there was no significant difference in the proportion of nulliparous meat-eaters and vegetarians who had undergone a hysterectomy (both 2%), but the proportion of parous meat-eaters who had previously undergone a hysterectomy remained double the proportion of parous vegetarians (12% vs 6%, $P = 0.02$).

### Associations between concentrations of oestradiol and SHBG and other descriptive variables

Amongst the premenopausal women, the strongest association was the significant decrease in SHBG concentration with both increasing weight and BMI (correlation coefficients − 0.32 and − 0.33 respectively; $P < 0.001$ for both). Among the post-menopausal women, the strongest associations were the significant increase in oestradiol concentration with both increasing weight and BMI (correlation coefficients 0.38 and 0.42; $P < 0.001$ for both), and the significant decrease in SHBG concentration with both increasing weight and BMI (correlation coefficients − 0.42 and − 0.49 respectively; $P < 0.001$ for both).

Premenopausal plasma concentrations of SHBG were significantly positively correlated with concentrations of oestradiol (correlation coefficient 0.27; $P < 0.001$). Post-menopausal plasma concentrations of oestradiol and SHBG were significantly negatively correlated, but after adjusting for BMI this association was no longer present (correlation coefficient = − 0.03; $P = 0.52$).

© 1999 Cancer Research Campaign

Both pre- and post-menopausal concentrations of SHBG were significantly positively correlated with HDL cholesterol (correlation coefficients 0.22 and 0.19; $P < 0.001$ for both), post-menopausal concentrations were also significantly positively associated with age at recruitment (correlation coefficient 0.12; $P = 0.01$). Neither oestradiol nor SHBG concentration was strongly associated with age at menarche, length of menstrual cycle (premenopausal women only), years past menopause, previous hysterectomy (post-menopausal women only), parity, hour of day of blood donation, or total and LDL cholesterol concentration.

### Hormone concentrations in the three dietary groups

Since concentrations of oestradiol (post-menopausal only) and SHBG were significantly associated with BMI, and since BMI varied significantly between dietary groups (Table 1), all subsequent analyses are presented with and without adjustment for BMI.

Amongst the premenopausal women, the mean oestradiol concentration was 2% lower in the vegetarians and vegans than in the meat-eaters, whilst the mean SHBG concentration was 3% higher in the vegetarians and 7% higher in the vegans than in the meat-eaters (Table 2). These results were adjusted for age and day of menstrual cycle; further separate adjustments for parity and hour of the day of blood sample collection had little effect on the results. Further adjustment for BMI had little effect on oestradiol, but reduced the difference in SHBG among the dietary groups. There was also no suggestion that there were differences in oestradiol concentration among dietary groups at different stages of the menstrual cycle. None of the differences in means was statistically significant.

Amongst the post-menopausal women, the mean oestradiol concentration was 4% lower in the vegetarians and 6% lower in the vegans than in the meat-eaters, whilst the mean SHBG concentration was 6% higher in the vegetarians and 12% higher in the vegans than in the meat-eaters. Adjusting for age, number of years past menopause, parity and hour of the day of blood sample collection had little effect on the results. After adjusting for BMI, the weak linear trends in oestradiol and SHBG concentrations across the three dietary groups were no longer present. The mean concentrations of oestradiol and SHBG (both unadjusted and adjusted for BMI) were also compared between dietary groups after excluding 34 post-menopausal women who had undergone a hysterectomy and two women for whom this information was missing. The mean concentrations in the three dietary groups were almost identical to those reported in Table 2.

### DISCUSSION

This study showed no statistically significant differences between meat-eaters, vegetarians and vegans in plasma concentrations of oestradiol or SHBG. Before adjusting for BMI there were small differences in the direction expected, with the vegetarians and vegans having higher SHBG and lower post-menopausal oestradiol than the meat-eaters. These small differences were essentially eliminated by adjusting for BMI. Thus this study implies that the relatively low BMI of vegetarians and vegans does cause small changes in SHBG and in post-menopausal oestradiol, but that the composition of vegetarian diets may not have any additional effects on these hormones.

In this study, women who ate meat had a greater mean BMI than women who followed a vegetarian or vegan diet, as reported in previous studies of vegetarians (Key and Davey, 1996; Appleby et al, 1998). The well-established (Potischman et al, 1996; Thomas et al, 1997*b*) negative associations between both pre- and post-menopausal SHBG concentration and BMI, and the strong positive association between post-menopausal oestradiol concentration and BMI were clearly demonstrated in these data. Insulin might mediate the obesity-related suppression of SHBG concentration since a negative correlation between SHBG and insulin levels has been reported (Franks et al, 1991). Obesity increases post-menopausal concentrations of oestradiol by increasing the aromatization of androstenedione to oestrone (which in turn is hydroxylated to oestradiol) in the peripheral adipose tissue (Siiteri and MacDonald, 1973; Judd et al, 1982).

Any association between a vegetarian diet and relatively low endogenous concentrations of oestradiol and relatively high concentrations of SHBG could simply be due to a relatively low BMI amongst vegetarians or might be due to the composition of the diet. A vegetarian or vegan diet is generally relatively low in saturated fat and high in dietary fibre in comparison to a diet that includes meat, and vegetarians have lower plasma total cholesterol concentrations than meat-eaters (Thorogood et al, 1987). Similarly, we observed that women who followed a vegetarian or vegan diet had significantly lower plasma concentrations of cholesterol, on average, than women who ate meat. Dietary fat intake might influence endogenous concentrations of oestradiol and SHBG via changes in BMI, whilst dietary fibre may interfere with the entero-hepatic cycling of oestrogens (Rose, 1990). In premenopausal women the absence of a strong dietary effect may be due to tight control of levels of oestradiol by feedback mechanisms.

Five studies have previously compared premenopausal plasma concentrations of oestradiol between women who have followed either a vegetarian or non-vegetarian diet for a relatively long period of time, but all were small, with the number of vegetarians in each study ranging from ten to 34 (Table 3). Three studies reported a non-significant difference in oestradiol concentration, ranging from a 19% lower to 13% higher plasma concentration of oestradiol in vegetarian women than in omnivorous women (Goldin et al, 1982; Gray et al, 1982; Shultz et al, 1987). Shultz and Leklem (1983) reported a significantly lower (by 30%) oestradiol concentration during the luteal phase in vegetarians than in meat-eaters, whilst Persky et al (1992) reported a non-significant, 26% lower luteal phase oestradiol concentration and a significantly 16% higher oestradiol concentration during the follicular phase in teenage vegetarians compared to omnivores. None of these studies found a statistically significant difference in BMI between the vegetarians and non-vegetarians, and none adjusted the results for BMI.

Only one study has compared post-menopausal plasma concentrations of oestradiol between long-term vegetarian and non-vegetarian women. Barbosa et al (1990) reported a 45% lower plasma oestradiol concentration in 12 vegetarians when compared to 12 non-vegetarian women. This difference in mean concentrations was statistically significant, but the results were not adjusted for the non-significant 9% lower BMI in the vegetarian women.

To our knowledge, only one study has compared plasma concentrations of SHBG between long-term vegetarian and non-vegetarian premenopausal women (Table 3). Fentiman et al (1988) reported a 3% lower mean concentration in 25 vegetarians than in 21 non-vegetarians. Three studies have previously investigated the differences in plasma SHBG concentration between long-term vegetarian and non-vegetarian post-menopausal women.

*© 1999 Cancer Research Campaign*

Armstrong et al (1981) reported a 16% higher concentration of SHBG (and a 4% lower mean BMI) in 43 vegetarians than in 44 non-vegetarians, whilst Barbosa et al (1990) reported a 12% lower mean SHBG concentration (and 9% lower mean BMI) in 12 vegetarians than in 12 non-vegetarians. Adlercreutz et al (1989) reported a 17% lower SHBG concentration in nine vegetarians than in ten non-vegetarians, which was consistent with the surprising 19% higher BMI in the vegetarians. None of the differences in mean concentration of SHBG was statistically significant and none of these studies adjusted their results for BMI.

A vegetarian diet is probably closer to the COMA recommendations for dietary intake (Department of Health, 1991) than any other easily defined dietary pattern (Resnicow et al, 1991), and if shown to have health benefits, it could be a Western diet with which a large proportion of the population could comply. A vegetarian or vegan diet does provide health advantages by lowering BMI, plasma cholesterol levels and mortality from ischaemic heart disease (Key et al, 1998). The most important modifiable determinant of oestradiol so far identified is BMI in post-menopausal women. Our results suggest that a vegetarian diet should cause a slight reduction in breast cancer risk in post-menopausal women by lowering BMI and therefore oestradiol; Key et al (1998) observed a non-significant 5% reduction in breast cancer mortality amongst vegetarians compared to non-vegetarians in five prospective studies. Further work is needed to establish whether specific dietary components are important determinants of oestradiol and, therefore, breast cancer risk.

### ACKNOWLEDGEMENTS

We thank all the participants of this study, the EPIC study staff at ICRF, Dr John Keenan and staff at the Biochemical Endocrinology laboratory at the Radcliffe Infirmary, Oxford (now at the John Radcliffe Hospital, Oxford), Professor Mitch Dowsett and staff at the Biochemical Endocrinology laboratory at the Royal Marsden Hospital, London, and the Clinical Biochemistry staff at Addenbrooke's Hospital, Cambridge. This study was supported by the Imperial Cancer Research Fund and by the Europe Against Cancer Programme of the Commission of the European Communities.

### REFERENCES

Adlercreutz H, Hamalainen E, Gorbach SL, Goldin BR, Woods MN and Dwyer JT (1989) Diet and plasma androgens in postmenopausal vegetarian and omnivorous women and postmenopausal women with breast cancer. *Am J Clin Nutr* **49**: 433–442

Appleby PN, Thorogood M, Mann JI and Key TJ (1998) Low body mass index in non-meat eaters: the possible roles of animal fat, dietary fibre and alcohol. *Int J Obesity* **22**: 454–460

Armstrong BK, Brown JB, Clarke HT, Crooke DK, Hahnel R, Masarei JR and Ratajczak T (1981) Diet and reproductive hormones: a study of vegetarian and non-vegetarian postmenopausal women. *J Natl Cancer Inst* **67**: 761–767

Barbosa JC, Shultz TD, Filley SJ and Nieman DC (1990) The relationship among adiposity, diet, and hormone concentrations in vegetarian and non-vegetarian postmenopausal women. *Am J Clin Nutr* **51**: 798–803

Department of Health (1991) *Dietary Reference Values for Food Energy and Nutrients for the United Kingdom*: *Report of the Panel on Dietary Reference Values, Committee on Medical Aspects of Food Policy*. Report on Health and Social Subjects No. 41. HMSO: London

Dowsett M, Goss PE, Powles TJ, Hutchinson G, Brodie AM, Jeffcoate SL and Coombes RC (1987) Use of the aromatase inhibitor 4-hydroxyandrostenedione in postmenopausal breast cancer: optimization of therapeutic dose and route. *Cancer Res* **47**: 1957–1961

Fentiman IS, Caleffi M, Wang DY, Hampson SJ, Hoare SA, Clark GMG, Moore JW, Bruning P and Bonfrer JMG (1988) The binding of blood-borne estrogens in normal vegetarian and omnivorous women and the risk of breast cancer. *Nutr Cancer* **11**: 101–106

Franks S, Kiddy DS, Hamilton Fairley D, Bush A, Sharp PS and Reed MJ (1991) The role of nutrition and insulin in the regulation of sex hormone binding globulin. *J Steroid Biochem Mol Biol* **39**: 835–838

Friedewald WT, Levy RI and Fredrickson DS (1972) Estimation of the concentration of low-density lipoprotein cholesterol in plasma, without use of the preparative ultracentrifuge. *Clin Chem* **18**: 499–502

Goldin BR, Adlercreutz H, Gorbach SL, Warram JH, Dwyer JT, Swenson L and Woods MN (1982) Estrogen excretion patterns and plasma levels in vegetarian and omnivorous women. *N Engl J Med* **307**: 1542–1547

Gray GE, Williams P, Gerkins V, Brown JB, Armstrong B, Phillips R, Casagrande JT, Pike MC and Henderson BE (1982) Diet and hormone levels in Seventh-Day Adventist teenage girls. *Prev Med* **11**: 103–107

Hankinson SE, Willett WC, Manson JE, Colditz GA, Hunter DJ, Spiegelman D, Barbieri RL and Speizer FE (1998) Plasma sex steroid hormone levels and risk of breast cancer in postmenopausal women. *J Natl Cancer Inst* **90**: 1292–1299

Judd HL, Shamonki IM, Frumar AM and Lagasse LD (1982) Origin of serum estradiol in postmenopausal women. *Obstet Gynecol* **59**: 680–686

Key T and Davey G (1996) Prevalence of obesity is low in people who do not eat meat [letter]. *Br Med J* **313**: 816–817

Key TJ, Chen J, Wang DY, Pike MC and Boreham J (1990) Sex hormones in women in rural China and in Britain. *Br J Cancer* **62**: 631–636

Key TJ, Fraser GE, Thorogood M, Appleby PN, Beral V, Reeves G, Burr ML, Chang-Claude J, Frentzel-Beyme R, Kuzma JW, Mann J and McPherson K (1998) Mortality in vegetarians and non-vegetarians: a collaborative analysis of 8300 deaths among 76 000 men and women in five prospective studies. *Public Health Nutr* **1**: 33–41

Persky VW, Chatterton RT, Van Horn LV, Grant MD, Langenberg P and Marvin J (1992) Hormone levels in vegetarian and non-vegetarian teenage girls: potential implications for breast cancer risk. *Cancer Res* **52**: 578–583

Pike MC, Spicer DV, Dahmoush L and Press MF (1993) Estrogens, progestogens, normal breast cell proliferation, and breast cancer risk. *Epidemiol Rev* **15**: 17–35

Potischman N, Swanson CA, Siiteri P and Hoover RN (1996) Reversal of relation between body mass and endogenous estrogen concentrations with menopausal status. *J Natl Cancer Inst* **88**: 756–758

Resnicow K, Barone J, Engle A, Miller S, Haley NJ, Fleming D and Wynder E (1991) Diet and serum lipids in vegan vegetarians: a model for risk reduction. *J Am Diet Assoc* **91**: 447–453

Riboli E and Kaaks R (1997) The EPIC Project: rationale and study design. *Int J Epidemiology* **26**: S6–S14

Rose DP (1990) Dietary fiber and breast cancer. *Nutr Cancer* **13**: 1–8

Shultz TD and Leklem JE (1983) Nutrient intake and hormonal status of premenopausal vegetarian Seventh-day Adventists and premenopausal non-vegetarians. *Nutr Cancer* **4**: 247–259

Shultz TD, Wilcox RB, Spuehler JM and Howie BJ (1987) Dietary and hormonal interrelationships in premenopausal women: evidence for a relationship between dietary nutrients and plasma prolactin levels. *Am J Clin Nutr* **46**: 905–911

Siiteri PK and MacDonald PC (1973) Role of extraglandular estrogens in human endocrinology. In *Handbook of Physiology*, Vol II, Part I, Geiger SR, Astwood EB and Greep RO (eds), pp. 615–629. American Physiological Society:Bethesda, MD

Siiteri PK, Hammond GL and Nisker JA (1981) Increased availability of serum estrogens in breast cancer: a new hypothesis. In *Hormones and Breast Cancer*, Pike MC, Siiteri PK, Welsch CW (eds), pp. 87–106. Cold Spring Harbor Laboratory: Cold Spring Harbor

Thomas HV, Reeves GK and Key TJA (1997*a*) Endogenous estrogen and postmenopausal breast cancer: a quantitative review. *Cancer Causes Control* **8**: 922–928

Thomas HV, Key TJ, Allen DS, Moore JW, Dowsett M, Fentiman IS and Wang DY (1997*b*) Reversal of relation between body mass and endogenous estrogen concentrations with menopausal status [letter]. *J Natl Cancer Inst* **89**: 396–398

Thorogood M, Carter R, Benfield L, McPherson K and Mann JI (1987) Plasma lipids and lipoprotein cholesterol concentrations in people with different diets in Britain. *Br Med J Clin Res Ed* **295**: 351–353

© 1999 Cancer Research Campaign 
